# Preparation and Stability Study of an Injectable Hydrogel for Artificial Intraocular Lenses

**DOI:** 10.3390/polym16182562

**Published:** 2024-09-10

**Authors:** Haifeng Cui, Pengfei Li, Zekun Su, Shiqiang Guan, He Dong, Xufeng Dong

**Affiliations:** 1 School of Materials Science and Engineering, Dalian University of Technology, Dalian 116024, China; 20211051180@mail.dlut.edu.cn (H.C.); 1589942884@mail.dlut.edu.cn (P.L.); suzekun1999@163.com (Z.S.); guanshiqiang@mail.dlut.edu.cn (S.G.); 2 Department Ophthalmology, The Third People’s Hospital of Dalian, Dalian 116033, China

**Keywords:** artificial intraocular lens, cataract treatment, hydrogel, tensile modulus, variable focus

## Abstract

Currently available intraocular lenses (IOLs) on the market often differ significantly in elastic modulus compared to the natural human lens, which impairs their ability to respond effectively to the tension of the ciliary muscles for focal adjustment after implantation. In this study, we synthesized a polyacrylamide–sodium acrylate hydrogel (PAH) through the cross-linking polymerization of acrylamide and sodium acrylate. This hydrogel possesses excellent biocompatibility and exhibits several favorable properties. Notably, the hydrogel demonstrates high transparency (94%) and a refractive index (1.41 ± 0.07) that closely matches that of the human lens (1.42). Additionally, it shows strong compressive strength (14.00 kPa), good extensibility (1400%), and an appropriate swelling ratio (50 ± 2.5%). Crucially, the tensile modulus of the hydrogel is 2.07 kPa, which closely aligns with the elastic modulus of the human lens (1.70–2.10 kPa), enabling continuous focal adjustment under the tension exerted by the ciliary muscles.

## 1. Introduction

Currently, an estimated 65 million people globally are affected by cataracts, with approximately 20 million of these individuals experiencing blindness as a direct consequence of the condition. This number is anticipated to increase as the global population continues to age. Despite the potential for cataracts to cause blindness, it is important to note that in most cases, vision can be effectively restored through surgical treatment. The growing prevalence of cataracts, driven by demographic shifts and increased life expectancy, underscores the need for widespread access to surgical interventions and eye care services to mitigate the impact of this condition on public health. Phacoemulsification combined with intraocular lens (IOL) implantation is widely regarded as the most effective treatment for cataracts globally [1,2]. The intraocular lenses currently available on the market can be broadly categorized into two types: monofocal and multifocal lenses. Both types have been shown to possess excellent biocompatibility, making them suitable for long-term use in patients [3,4,5]. Despite these advantages, a significant limitation of these IOLs is their inability to provide continuous focal adjustment. This limitation means that they cannot fully address the diverse visual needs of all patients, particularly those who require a broader range of focus for activities at varying distances [6,7,8,9].

An ideal intraocular lens (IOL) material should exhibit superior optical and mechanical properties, excellent biocompatibility, and ideally, be suitable for injection-based implantation into the human eye. Such a material would need to rapidly solidify with minimal exothermic reaction during the process, thereby minimizing surgical incisions and reducing tissue damage [10,11,12]. Currently, the IOLs used in clinical practice include the following: (1) Acrylic IOLs, which are available in several forms, including hydrophilic, hydrophobic, and hydrophilic types with hydrophobic surface treatments [13,14,15]. Polymethyl methacrylate (PMMA) has become one of the most widely utilized materials in IOL manufacturing due to its excellent biocompatibility, stable performance, and relatively low cost. However, its elastic modulus, ranging from 2.11 to 3.42 GPa, makes it non-foldable, which limits its use in phacoemulsification procedures and necessitates larger surgical incisions [16,17,18]. (2) Silicone IOLs, which are composed of polydimethylsiloxane and dimethylvinylsiloxane terminal groups [19,20]. These lenses offer effective postoperative vision correction, but their elastic modulus, ranging from 1.13 to 3.04 MPa, still imposes certain limitations [21,22,23,24]. The key drawback of both these non-variable focus IOL materials is their inability to adjust focal length rapidly in response to changes in viewing distance, which significantly limits the overall visual performance for patients [25,26,27,28,29,30].

PAH has good biocompatibility; previous studies have conducted biosafety assessments of this hydrogel in relation to the human eye. The results indicate that this hydrogel exhibits good biocompatibility, does not induce cellular inflammation, and maintains favorable biocompatibility when implanted in rabbit eyes [31]. The PAH is primarily composed of acrylamide and sodium acrylate as its main raw materials, and the polyacrylamide–sodium acrylate hydrogel (PAH) was synthesized using tetramethylethylenediamine as the initiator [32,33,34,35]. Hydrogel samples were then fabricated using appropriate molds to meet the experimental requirements [36,37]. In this study, we will evaluate the surface morphology, structure, mechanical properties, optical properties, and swelling behavior of the PAH. Additionally, the elastic modulus and zooming capability of the hydrogel will be tested and assessed, with a discussion on its potential advantages as a novel material for variable-focus intraocular lenses.

The novelty of the new adjustable-focus intraocular lens lies in its departure from the fixed focal point of traditional intraocular lenses, introducing the concept of mimicking the zooming mechanism of the human eye lens in its design. The testing methods and results presented in this paper will provide a comprehensive validation of the innovation and practicality of this new intraocular lens [38,39,40,41,42,43].

## 2. Materials and Methods

### 2.1. Materials

The materials used in the preparation are as follows: Acrylamide (Acrylamide, Shanghai Macklin Biochemical Co., Ltd., Shanghai, China), Sodium Acrylate (Sodium Acrylate, Shanghai Macklin Biochemical Co., Ltd., Shanghai, China), N,N′-Methylenebisacrylamide (MBA, Shanghai Macklin Biochemical Co., Ltd., Shanghai, China), Ammonium Persulfate (APS, Shanghai Macklin Biochemical Co., Ltd., Shanghai, China), Tetramethylethylenediamine (TEMED, Shanghai Macklin Biochemical Co., Ltd., Shanghai, China), Phosphate Buffered Solution (PBS, Shanghai Macklin Biochemical Co., Ltd., Shanghai, China), Calcium Nitrate Tetrahydrate Solution (Shanghai Macklin Biochemical Co., Ltd., Shanghai, China).

### 2.2. Preparation of Polyacrylamide–Sodium Acrylate Hydrogels

The polyacrylamide–sodium acrylate hydrogel was prepared using acrylamide and sodium acrylate as primary raw materials, methylene bisacrylamide as a cross-linker, ammonium persulfate as an initiator, and tetramethylethylenediamine as a catalyst through copolymerization in an aqueous medium.

**Figure 1:**
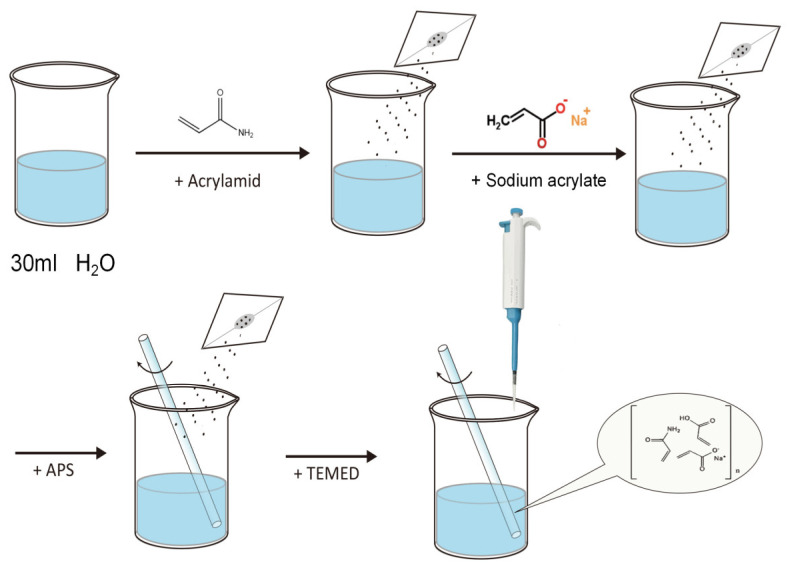
Preparation process of PAH.

The preparation process of PAH is illustrated in Figure 1. First, the primary raw materials, acrylamide (87% molar ratio) and sodium acrylate (12.5% molar ratio), are thoroughly mixed, and water is added with stirring to ensure complete dissolution. Subsequently, N,N′-methylenebisacrylamide (0.5% molar ratio) and a 10% ammonium persulfate solution are added, followed by stirring for 5 min to dissolve all the ingredients. After the thorough mixing of the aforementioned materials, a pipette is used to add the catalyst tetramethylethylenediamine, and the mixture is left to stand for 15 min to allow complete solidification. Table 1 presents the composition of the raw materials.

### 2.3. Characterization

The lyophilized hydrogel samples were first cryofractured in liquid nitrogen to obtain brittle fracture surfaces, followed by drying in a freeze dryer (Lab-1-50, Boyikang Experimental Instrument Co., Ltd., Beijing, China) for 48 h to ensure complete dehydration. After gold sputter-coating, the morphology of the samples was examined using a laser confocal microscope (LSM780, Zeiss, Jena, UK) and a high-resolution field emission scanning electron microscope (SEM, IT800-SHL, JEOL, Tokyo, Japan) under an accelerating voltage of 10 kV. This comprehensive approach allowed for detailed observation and analysis of the hydrogel’s surface characteristics.

### 2.4. Fourier Transform Infrared Spectroscopy Analysis

Fourier transform infrared spectroscopy (FTIR) was employed using an IS50 spectrometer (Thermo Fisher Scientific, Waltham, MA, USA) to investigate the chemical bonding structure within PAH and to verify the completion of the reaction. The PAH samples were first frozen at −20 °C for 12 h to immobilize the water content within the hydrogel network. Subsequently, the samples were freeze-dried for 48 h using a vacuum freeze dryer to obtain lyophilized PAH samples. The freeze-dried samples were then ground into powder using a mortar and pestle and analyzed via infrared spectroscopy in comparison with the raw materials, SA and AM.

### 2.5. Thermal Stability Testing

The thermal properties of hydrogels determine their stability across different temperature environments. Since the internal temperature of the human body is typically maintained at around 37 °C, biomaterials must retain their structure and function within this temperature range without excessive swelling, shrinkage, or degradation. Additionally, thermal properties influence the phase transition behavior of hydrogels, with the associated energy changes being particularly crucial for their safety. Appropriate thermal properties ensure that biomaterials do not fail due to temperature fluctuations, thereby providing long-term safety and efficacy.

To investigate the thermal stability of PAH, a differential scanning calorimeter (DSC3, Mettler-Toledo, Greifensee, Switzerland) was used to obtain the phase change latent heat DSC curve of the hydrogel. A 5.4 mg sample of PAH was sealed in an aluminum crucible and heated in a pure nitrogen atmosphere at a rate of 2 °C/min over a temperature range of 0 to 50 °C. Additionally, thermogravimetric analysis (TGA) was performed using a simultaneous thermal analyzer (TGA/DSC3+, Mettler-Toledo, Switzerland) from room temperature up to 325 °C under a nitrogen environment.

### 2.6. Tensile Performance Testing

Good tensile properties mean that the hydrogel can maintain its integrity during stretching or deformation, making it resistant to breaking or damage. This property is particularly important for applications like intraocular lenses, which must withstand the tension of the suspensory ligaments over long periods. Adequate tensile strength ensures that the hydrogel possesses sufficient durability and flexibility within the body, meeting biomechanical requirements and preventing material failure or tissue damage.

Hydrogel samples were tested using a universal tensile testing machine (HY-0580, Shanghai Hengyi Precision Instruments Co., Ltd., Shanghai, China). The hydrogels are cut into dumbbell shapes according to the national standard GB/T-528-2009 [44], with a tensile rate of 50 mm/min.

### 2.7. Compression Performance Testing

The human eye maintains stable intraocular pressure and may also be subjected to external pressures. As a material for intraocular lenses, appropriate compressive properties ensure that the hydrogel can effectively distribute pressure within the eye, providing necessary support and protection. This helps prevent material failure or damage to surrounding tissues.

The hydrogel samples were tested using a universal tensile testing machine (HY-0580, Shanghai Hengyi Precision Instrument Co., Ltd., China). According to the national standard GB/T 9341-2000 [45], the hydrogels were cut into cylindrical specimens with a height of 7.45 mm and a diameter of 14.6 mm using a cutter conforming to the national standard HG/T 2645-2011 [46]. The compression rate was set at 50 mm/min, and five compression tests were conducted.

### 2.8. Fatigue Resistance Test

Robust fatigue resistance is essential for an intraocular lens, as it must endure cyclic tensile and compressive forces over an extended duration. Should the fatigue resistance be insufficient, the hydrogel may succumb to fracturing, degradation, or failure under repeated loading, thereby undermining its reliability and safety in biomedical applications.

According to the GB/T 9341-2000 standard, cylindrical hydrogel samples with a height of 10 mm and a diameter of 10 mm were prepared using a cutter compliant with the national standard HG/T 2645-2011. The samples were subjected to six compression cycles within 6 s using a universal tensile testing machine (HY-0580, Shanghai Hengyi Precision Instrument Co., Ltd., China). The fatigue resistance of the hydrogel was evaluated by observing changes in the cyclic compression curves.

### 2.9. Transparency Testing

In accordance with the national standard GB/T 1041-92 [47], hydrogel samples were prepared in 10 mm cubic forms using a cutter compliant with the national standard HG/T 2645-2011. The samples were tested using a UV transmittance analyzer (ND-1000, Changchun Kexin Laboratory Instrument Co., Ltd., Changchun, China). The transmittance was recorded over the wavelength range of 450 nm to 900 nm, and a transmittance curve was plotted.

### 2.10. Refractive Index Testing

The human eye constitutes a sophisticated optical system, wherein light traverses multiple strata of media before forming an image on the retina. This necessitates that each layer possesses a refractive index harmonized with the overall system. As a surrogate for the lens, which serves as the eye’s principal refractive organ, the hydrogel must exhibit a highly specific refractive index. Consequently, it is imperative to conduct precise measurements of the hydrogel’s refractive index.

In accordance with the national standard GB/T 1041-92, hydrogel samples were prepared as 10 mm cubic specimens using a cutter compliant with the national standard HG/T 2645-2011. Incident and emergent angles were measured to calculate refractive index (*n*) using the following formula:

(1) $$n=\frac{\sin\alpha }{\sin\beta }$$

### 2.11. Focal Length Measurement

The calculated refractive index and the paraxial imaging formula are used for single refractive spherical surfaces:

(2) $$N-\frac{n}{r}=\frac{n}{s}+\frac{N}{S}$$

In this context, *s* represents the object distance, *S* denotes the image distance, and *r* is the radius of curvature. The resulting *N* corresponds to the theoretical focal length. The measured data were subsequently compared with the actual focal length to assess the accuracy of the theoretical model.

### 2.12. Zoom Performance Testing

In the human eye, the lens changes its surface curvature in response to the tension applied by the ciliary muscles and suspensory ligaments, enabling the adjustment of focal length. This process is central to the eye’s ability to focus. To validate the zooming capability of an artificial lens, it is similarly necessary to induce shape changes in the hydrogel to measure the corresponding variations in focal length.

**Figure 2:**
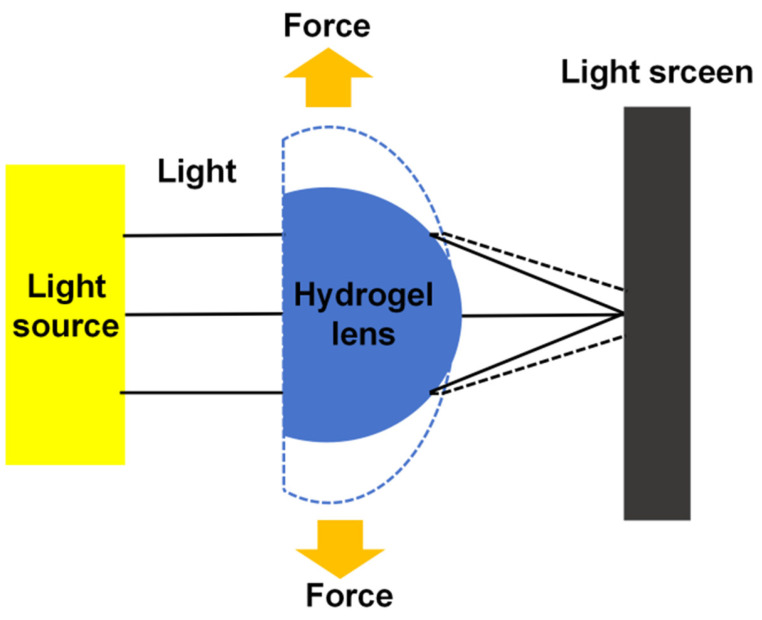
Schematic diagram of focal length measurement of PAH.

The deformation of the hydrogel was induced by applying external tensile stress, simulating the stretching process of the ciliary muscles. To evaluate the optical behavior of the hydrogel under varying stress conditions, a laser pointer was employed to illuminate the sample, generating a point light source. This setup allowed for the precise measurement of changes in the optical path and focal length as the applied stress varied, providing insights into the hydrogel’s response to different mechanical loads, as depicted in Figure 2.

### 2.13. Swelling Performance Testing

Swelling performance refers to the ability of hydrogels to expand in volume after absorbing water. For hydrogels used in human tissues, the swelling performance is of critical importance. The hydrogel must exhibit sufficient swelling to absorb water and remain soft, but excessive swelling must be avoided as it may cause tissue compression or dislocation. Optimal swelling performance ensures that the hydrogel remains stable within the body.

To investigate the effect of poly(allylamine hydrochloride) (PAH) on the swelling capacity of hydrogels in different concentration solutions, an experiment was designed with three groups: distilled water, 5× PBS solution, and 10× PBS solution. Small cubic hydrogel samples with edge lengths of 10 mm were used to assess swelling performance. The swelling behavior was observed and recorded every 3 h for a total duration of 72 h.

### 2.14. Rheological Performance Testing

The appropriate balance between loss modulus and storage modulus ensures that the hydrogel possesses enough flexibility to adapt to the dynamic environment within the body, while avoiding excessive energy dissipation that could compromise its performance. This balance not only extends the hydrogel’s service life but also minimizes its impact on surrounding tissues. Furthermore, by analyzing the loss modulus and storage modulus, the potential instability of the hydrogel under varying amplitude conditions can be assessed. This enables the identification of the critical amplitude at which the gel becomes unstable, helping to determine whether the hydrogel remains safe under typical operating conditions.

Dynamic viscoelasticity was tested using a rotational rheometer (MCR301, Anton Paar, Graz, Austria), with hydrogel disks of 13 mm diameter and 2 mm thickness at 25 °C and 37 °C, under 1 Hz frequency and 0.01–100% strain amplitude, recording storage modulus (*G*′) and loss modulus (*G*″) to calculate loss angle (*δ*):

(3) $$tan\;\delta =\frac{G\prime }{G\prime \prime }$$

## 3. Results and Discussion

### 3.1. Hydrogel Characterization

**Figure 3:**
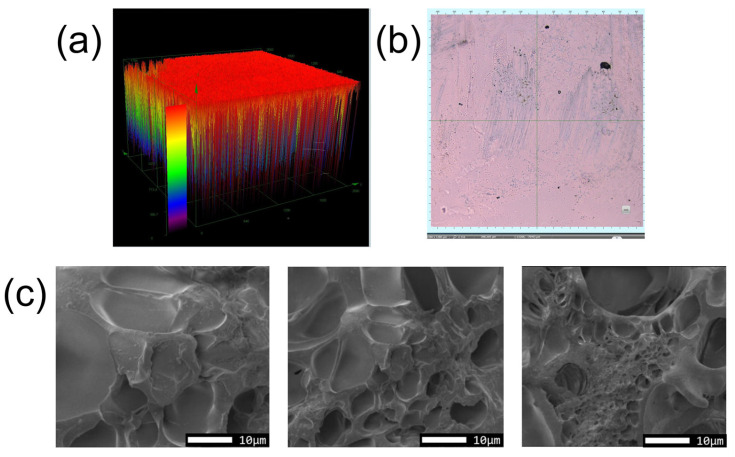
(**a**) Three-dimensional schematic diagram of PAH under laser confocal microscopy; (**b**) surface observation of PAH under laser confocal microscopy; (**c**) scanning electron microscope view of PAH.

**Figure 4:**
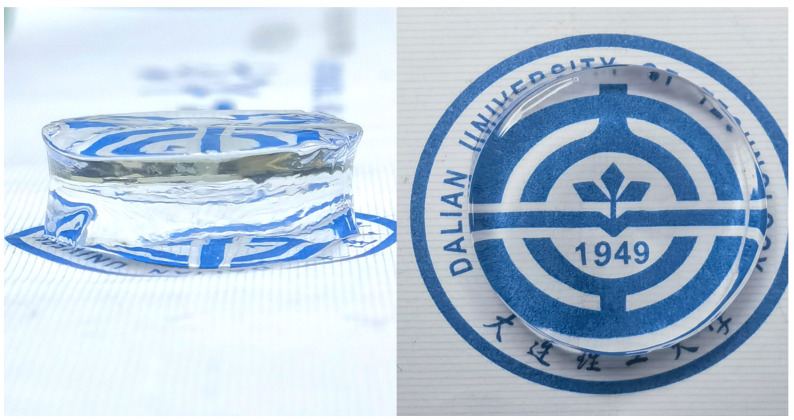
Schematic diagram of the optical transparency of PAH.

Figure 3 shows the laser confocal 3D images and SEM observations of the hydrogel, revealing smooth and porous structures. The porous structure allows the hydrogel to better disperse the stress when subjected to external forces, thereby improving its toughness and strength. Figure 4 shows a photograph of a sample of the PAH with excellent light transmission.

### 3.2. FTIR Analysis Results

**Figure 5:**
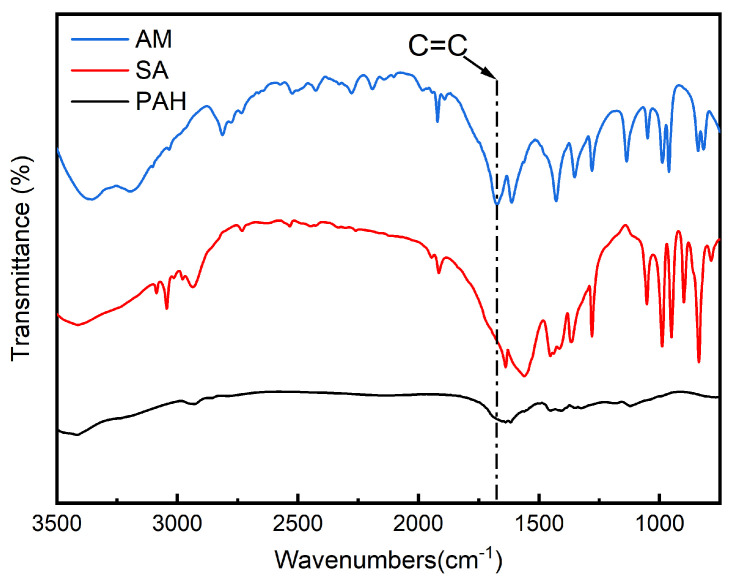
FTIR spectra of PAH.

To confirm the complete reaction of the raw materials, a comparative analysis of the FTIR spectra of AM, SA, and PAH was conducted, as shown in Figure 5. The FTIR spectra comparison between PAH and the reactants revealed an absorption peak at 1750 cm^−1^ in the AM spectrum, corresponding to the C=C bond present in AM. During the polymerization reaction, this C=C bond undergoes addition polymerization and should, therefore, disappear in the PAH spectrum. The absence of this absorption peak in the PAH spectrum confirms that the C=C bonds in AM have been fully reacted, indicating a complete copolymerization of AM and SA, leading to the formation of a stable polymer.

### 3.3. Thermal Stability Analysis

**Figure 6:**
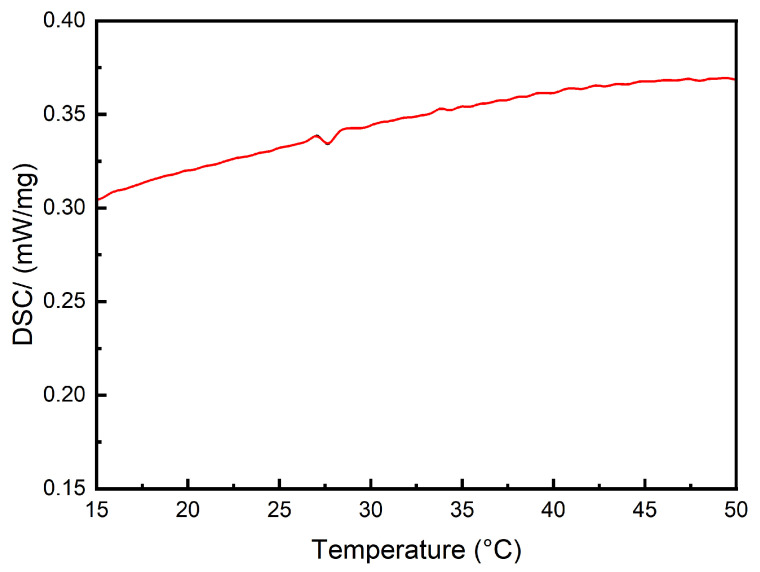
The DSC curve of PAH.

**Figure 7:**
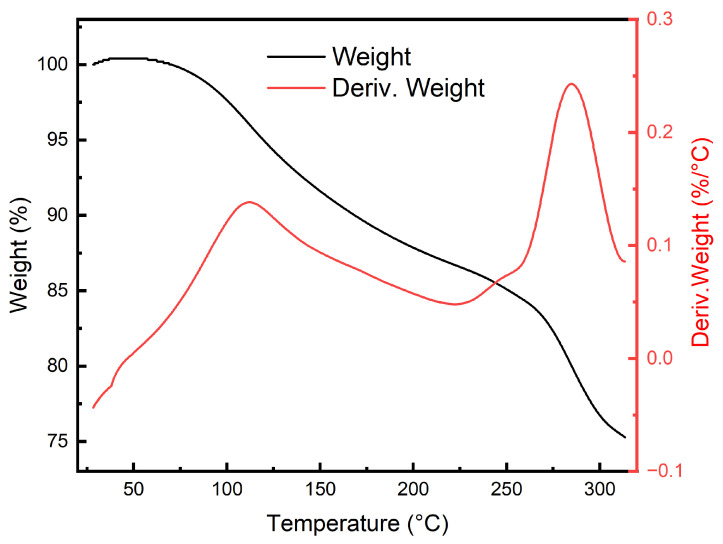
TGA and DTGA curves of PAH.

The DSC curve in Figure 6 indicates that PAH exhibits significant stability within the normal temperature range, with only a phase transition peak observed at 27.5 °C. The latent heat of the transition is −0.1227 J/g, and the heat flow is measured at 0.006454 mW/mg, indicating minimal heat release, well within the tolerance limits of the human eye. In the TGA and DTGA analyses shown in Figure 7, it can be observed that the phase transition identified in the DSC curve has a negligible impact on the mass of the material. The decomposition of PAH is initiated at 75 °C, and even as the temperature increases to 300 °C, the weight retention remains at 75%. The DTGA analysis reveals a marked increase in the decomposition rate near 100 °C and 270 °C, corresponding to the loss of water molecules and the decomposition of polyacrylamide, respectively. However, at the operational temperature of the hydrogel, around 37 °C, water molecules remain tightly bound within the gel network, preventing thermal decomposition. Consequently, this hydrogel demonstrates exceptional thermal stability, making it an ideal candidate for use as an artificial lens.

### 3.4. Tensile Performance

**Figure 8:**
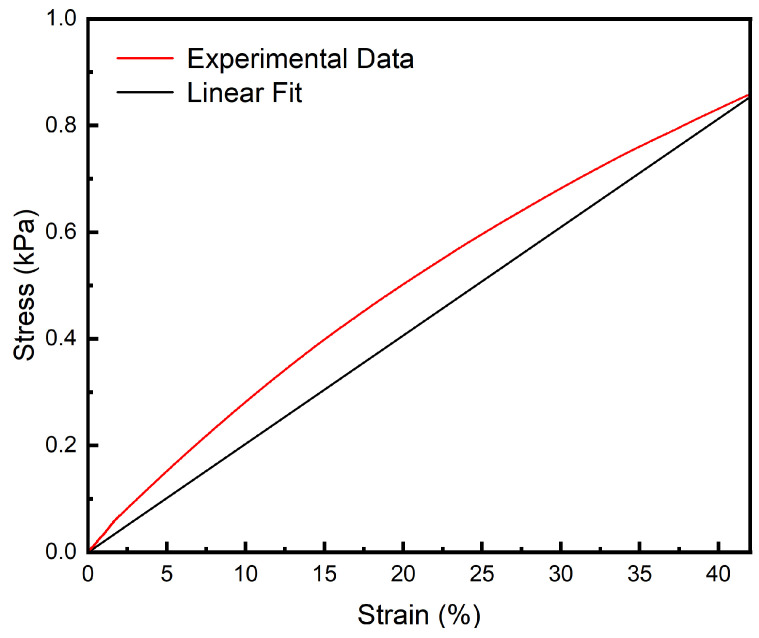
Tensile curve of the PAH.

The human eye lens deforms under the tension exerted by the zonular fibers, typically by no more than 40% of its original shape. As shown in Figure 8, within this strain range, the hydrogel can withstand a maximum tensile stress of 0.80 kPa without experiencing material failure. The tensile elastic modulus of the hydrogel at 40% deformation is measured at 2.07 kPa, which closely matches the elastic modulus of the natural human lens. This close alignment suggests that the hydrogel possesses both the necessary tensile strength and elasticity required to mimic the mechanical behavior of the human lens. Additionally, it demonstrates that the hydrogel can endure the required tensile stress without compromising its structural integrity. This is crucial for ensuring that the artificial lens remains securely anchored within the capsular bag after implantation, thereby maintaining its stability and function following surgery.

### 3.5. Compression Performance

**Figure 9:**
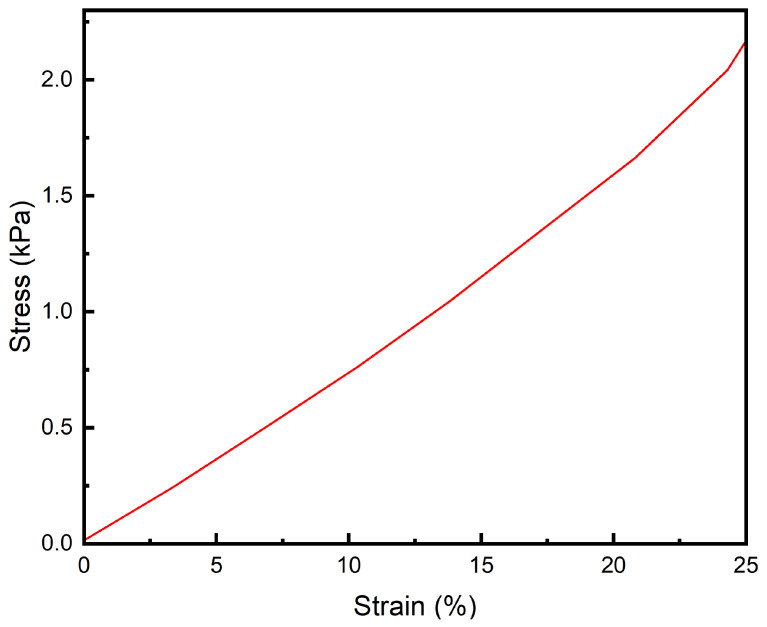
Compression curve of the PAH.

As can be seen from Figure 9, polyacrylamide–sodium acrylate hydrogels can withstand pressures greater than 2.0 kPa, and the intraocular pressure of the human eye is typically 1.3–2.0 kPa. It can be seen that PAH can withstand the intraocular pressure of the human eye and can maintain good stability.

### 3.6. Fatigue Resistance Analysis

**Figure 10:**
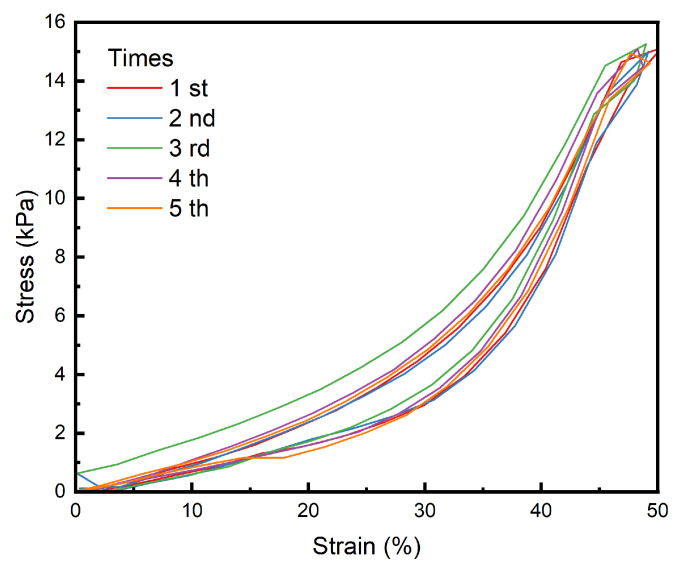
Test curve of PAH fatigue resistance.

The curve shown in Figure 10 was obtained by repeated compression of the hydrogel sample, and it can be seen that the hydrogel has stable performance and good fatigue resistance in a short period of time and multiple compressions.

### 3.7. Transparency Analysis

**Figure 11:**
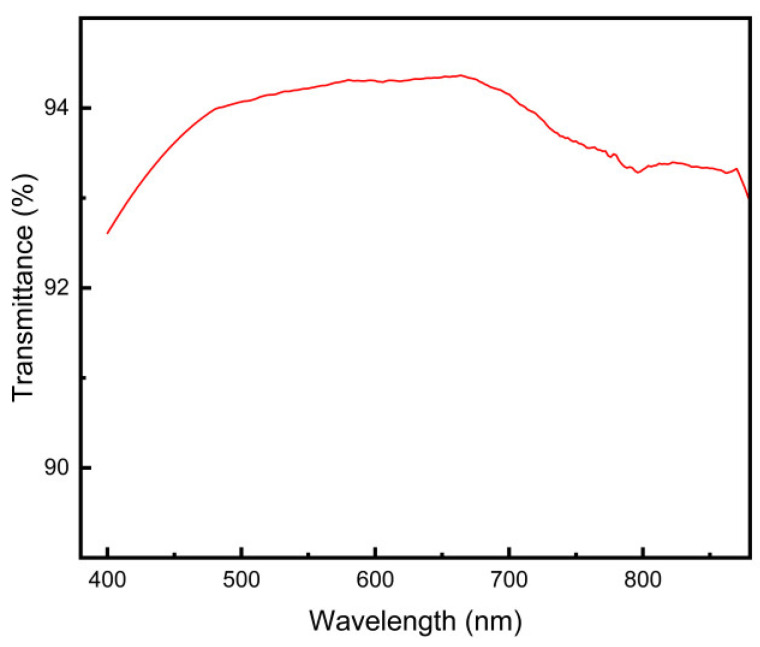
Light transmittance test curves of PAH.

By recording the transmittance in the 450–900 nm band, the transmittance curve was plotted in this paper as shown in Figure 11. As can be seen from Figure 11, the transmittance of the hydrogel is about 94%, the transmittance of the human eye lens is about 87%, and the transmittance of the polyacrylamide–sodium acrylate hydrogel can well meet the needs, and the prepared intraocular lens will have a better visual effect in clinical practice [48].

### 3.8. Refractive Index Analysis

In this paper, four groups of incidence angles a(°) and exit angles b(°) were recorded by fitting and modeling the optical path, and the refractive index n was calculated. According to the fitted 4 optical paths, the data obtained are shown in Table 2, and it can be seen that the refractive index of the PAH is about 1.415, which is close to the refractive index of the lens of the human eye. Therefore, the refractive index of polyacrylamide–sodium acrylate can meet the requirements of the lens of the human eye [49].

### 3.9. Focal Length Measurement

Based on the paraxial approximation formula for the on-axis configuration of a uniaxial spherical lens, the hydrogel with a curvature radius of 5 mm—which is within the typical range for the human eye lens (5 to 6 mm)—is calculated to have a focal length of approximately 18.2 mm. This value is very close to the focal length of the human eye lens, which is around 17.8 mm. Upon actual measurement, the focal length of the hydrogel was found to be 18.5 mm, demonstrating a close agreement with both the theoretical calculation and the actual focal length of the human eye lens. This consistency between the measured and calculated values further validates the hydrogel’s potential as a suitable material for mimicking the optical properties of the human lens.

### 3.10. Zoom Performance Analysis

**Figure 12:**
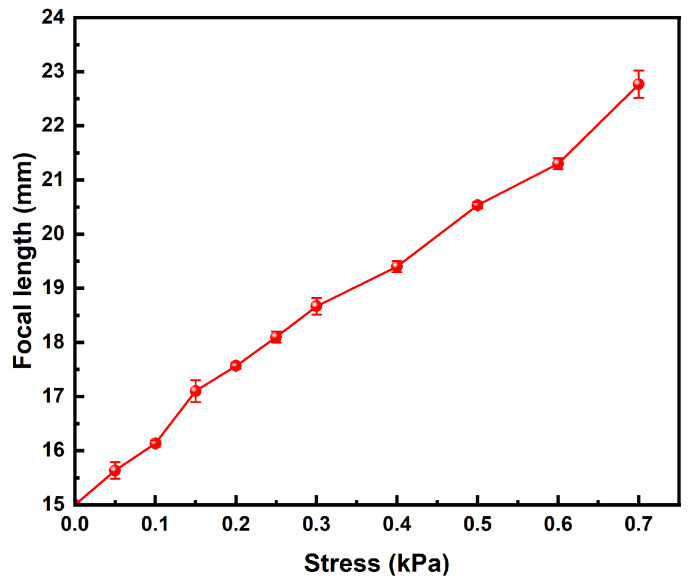
Test curves for zoom performance of PAH.

In the experiment, the tensile modulus of the hydrogel was found to closely match that of the human eye lens. When a variable tensile force was applied to the hydrogel using a universal tensile testing machine, the PAH exhibited noticeable deformation under tension. This deformation was accompanied by changes in the focal length, corresponding to the alterations in surface curvature. The results presented in Figure 12 were obtained through precise control of the applied tensile stress. To further analyze the optical behavior of the hydrogel, the sample was illuminated with a point light source. The resulting changes in the optical path were carefully recorded, allowing for the measurement of variations in both the refractive index and focal length of the hydrogel under different levels of tensile strain. This comprehensive approach provided detailed insights into how the hydrogel’s mechanical deformation influences its optical properties, closely simulating the dynamic focusing ability of the human lens.

According to the data in Figure 12, it can be seen that the focal length of the hydrogel can be observed to change significantly by adjusting the tensile stress applied to the hydrogel, which shows that the PAH has good zoom performance.

### 3.11. Swelling Performance

**Figure 13:**
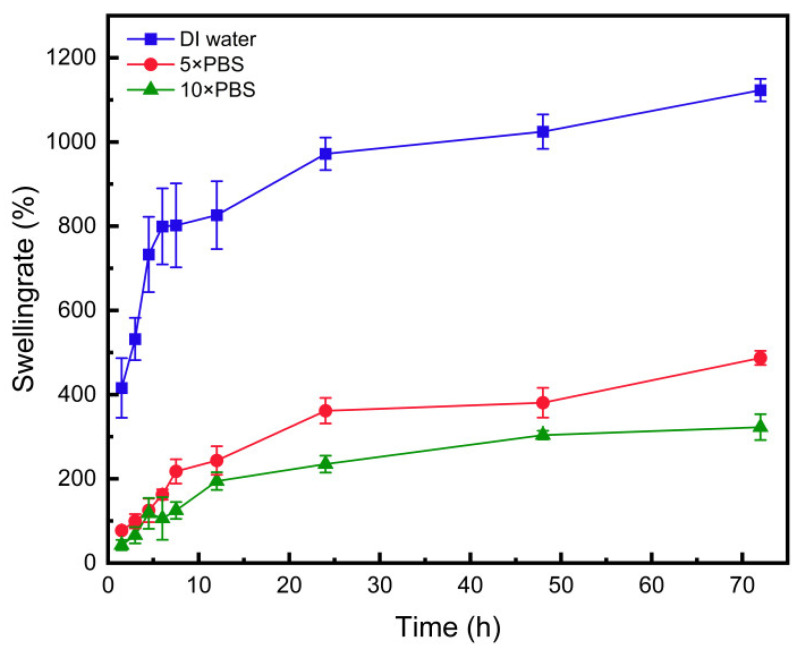
Test curves of swelling properties of PAH.

By recording the changes in the edge length of hydrogel cubes over time in Groups 1, 2, and 3, we derived the swelling curves of the hydrogel in different solutions. Analysis of the collected data from these groups revealed that the hydrogel in the distilled water group exhibited the fastest swelling rate and the most significant increase in volume. In contrast, the hydrogel in the 10× PBS solution group displayed the slowest swelling rate, with its final volume stabilizing at approximately twice its initial size. As shown in Figure 13, there is a clear trend where increasing the concentration of PBS solution leads to a reduction in the final swollen volume of the hydrogel, as well as a slower swelling rate. Given that the composition and concentration of aqueous humor in the human eye are similar to those of 10× PBS, it can be inferred that the hydrogel would swell appropriately and maintain stability when exposed to aqueous humor. This behavior is critical for ensuring the long-term performance and reliability of the hydrogel when used in ophthalmic applications, particularly as an intraocular lens material.

### 3.12. Heological Performance

**Figure 14:**
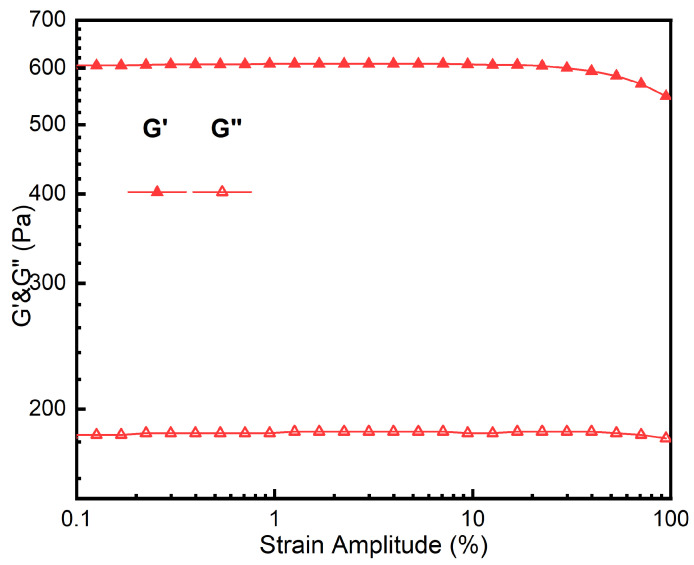
Test curves of storage modulus and loss modulus of PAH.

After the shock test, the G′ and G″ of the hydrogel at two temperatures were obtained, as shown in Figure 14. The G′ and G″ of the hydrogel remained stable, indicating that the deformation was in a linear form in this range.

The storage modulus and energy dissipation modulus of hydrogels are important parameters to reflect their state. When the storage modulus is greater than the energy dissipation modulus, the hydrogel behaves as a solid phase. When the storage modulus is less than the energy dissipation modulus, the hydrogel behaves as a fluid phase.

**Figure 15:**
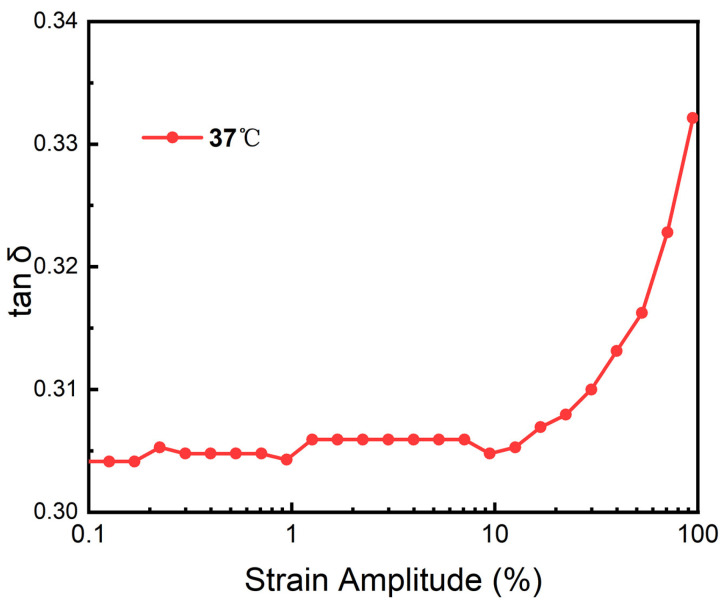
Calculation curve of loss angle of PAH.

The results of Figure 15 show that the loss factor remains relatively constant before the 10% strain, and there is no drastic fluctuation, which proves that the internal structure of the hydrogel remains stable during this process. When the strain amplitude is greater than 10%, the loss factor gradually increases, and the hydrogel begins to be unstable. In the process of changing the overall strain amplitude, the loss factor is always less than 0.4, which proves that the hydrogel has excellent elasticity. In daily life, the working environment of the lens is relatively stable, and the amplitude of more than 10% will not occur. Meanwhile, it is also a solid phase that can maintain its own form and meet the conditions for being an intraocular lens.

## 4. Conclusions

The polyacrylamide–sodium acrylate hydrogel was prepared using acrylamide and sodium acrylate as primary raw materials, methylene bisacrylamide as a cross-linker, ammonium persulfate as an initiator, and tetramethylethylenediamine as a catalyst through copolymerization in an aqueous medium. FTIR spectroscopy confirmed the reaction’s completeness, showing no unreacted components. CLSM and SEM analyses revealed a smooth surface and porous internal structure, providing effective water retention and some adhesiveness.

The hydrogel exhibited high transparency and a refractive index close to that of the human eye lens, indicating strong potential for artificial intraocular lenses (IOLs). Mechanical testing showed substantial tensile and compressive strength, excellent fatigue resistance, and consistent solid-phase behavior. The hydrogel’s elastic modulus of 2.07 kPa, similar to the human lens, enables it to adjust surface curvature under zonular tension, critical for variable focus in IOLs.

The hydrogel also demonstrated robust swelling stability, ceasing to swell after 72 h in 10× PBS solution, and maintained thermal stability between 15–40 °C. These properties suggest the hydrogel is biocompatible and suitable for long-term use, especially in minimally invasive injectable procedures, where it can solidify in situ, potentially reducing postoperative inflammation and improving patient outcomes.

## Figures and Tables

**Table 1:** Ingredients of PAH.

Number	Material	Molar Mass (g/mol)	Molar Ratio (%)	Concentration (%)	Volume (μL)
1	Acrylamide	71.08	87	30	55.61
2	Sodium Acrylate	94.04	12.5	20	15.86
3	MBA	154.2	0.5	2	10.4
4	TMEDA	116.2	-	-	0.3
5	APS	228.2	-	10	1
6	Water	-	-	-	118.13

**Table 2:** Measurement of the refractive index of PAH.

Number	Angle of Incidence (°)	Angle of Reflection (°)	Refractive Index
1	23.446	102.538	1.416
2	19.576	86.186	1.413
3	25.307	110.320	1.417
4	26.835	117.150	1.412

## Data Availability

The original contributions presented in the study are included in the article, further inquiries can be directed to the corresponding author.

## References

[1] Cooper R.C., Yang H. (2019). Hydrogel-based ocular drug delivery systems: Emerging fabrication strategies, applications, and bench-to-bedside manufacturing considerations. J. Control. Release.

[2] Cheng J.-W., Wei R.-L., Cai J.-P., Xi G.-L., Zhu H., Li Y., Ma X.-Y. (2007). Efficacy of different intraocular lens materials and optic edge designs in preventing posterior capsular opacification: A meta-analysis. Arch. Ophthalmol..

[3] Kim J., Dunn A.C. (2016). Soft hydrated sliding interfaces as complex fluids. Soft Matter.

[4] Li X., Sun Q., Li Q., Kawazoe N., Chen G. (2018). Functional hydrogels with tunable structures and properties for tissue engineering applications. Front. Chem..

[5] Özyol P., Özyol E., Karel F. (2017). Biocompatibility of intraocular lenses. Turk. J. Ophthalmol..

[6] Tong Z., Jin L., Oliveira J.M., Reis R.L., Zhong Q., Mao Z., Gao C. (2020). Adaptable hydrogel with reversible linkages for regenerative medicine: Dynamic mechanical microenvironment for cells. Bioact. Mater..

[7] Zare M., Bigham A., Zare M., Luo H., Rezvani Ghomi E., Ramakrishna S. (2021). Phema: An overview for biomedical applications. Int. J. Mol. Sci..

[8] Hu X., Grinstaff M.W. (2023). Advances in hydrogel adhesives for gastrointestinal wound closure and repair. Gels.

[9] Xia Y., Liu H., Shi S., Chen X., Jiao S. (2020). Preparation and properties of poly(acrylic ester) hydrogel as a potential intraocular lens for cataract treatment. Pharm. Nanotechnol..

[10] Li X., Zhao Y., Wang K., Wang L., Yang X., Zhu S. (2017). Cyclodextrin-containing hydrogels as an intraocular lens for sustained drug release. PLoS ONE.

[11] Han F., Wang T., Liu G., Liu H., Xie X., Wei Z., Li J., Jiang C., He Y., Xu F. (2022). Materials with tunable optical properties for wearable epidermal sensing in health monitoring. Adv. Mater..

[12] Sommer A.C., Blumenthal E.Z. (2019). Implementations of 3D printing in ophthalmology. Graefe’s Arch. Clin. Exp. Ophthalmol..

[13] Lee H., Oh H.J., Yoon K.C., Tae G., Kim Y.H. (2013). Fast in situ enzymatic gelation of PPO-PEO block copolymer for injectable intraocular lens in vivo. J. Biomater. Appl..

[14] Singh B., Dhiman A., Rajneesh K.A. (2016). Slow release of ciprofloxacin from β-cyclodextrin containing drug delivery system through network formation and supramolecular interactions. Int. J. Biol. Macromol..

[15] De Groot J.H., Spaans C.J., van Calck R.V., van Beijma F.J., Norrby S., Pennings A.J. (2003). Hydrogels for an accommodating intraocular lens. An explorative study. Biomacromolecules.

[16] Zhang Y., Ren K., He Z., Li H., Chen T., Lei Y., Xia S., He G., Xie Y., Zheng Y. (2013). Development of inclusion complex of brinzolamide with hydroxypropyl β-cyclodextrin. Carbohydr. Polym..

[17] Bozukova D., Pagnoulle C., De Pauw-Gillet M.C., Desbief S., Lazzaroni R., Ruth N., Jérôme R., Jérôme C. (2007). Improved performances of intraocular lenses by poly (ethylene glycol) chemical coatings. Biomacromolecules.

[18] Wielders L.H., Lambermont V.A., Schouten J.S., Biggelaar F.J.v.D., Worthy G., Simons R.W., Winkens B., Nuijts R.M. (2015). Prevention of cystoid macular edema after cataract surgery in nondiabetic and diabetic patients: A systematic review and meta-analysis. Arch. Ophthalmol..

[19] Shin M.K., Ji Y.W., Moon C.E., Lee H., Kang B., Jinn W.S., Ki J., Mun B., Kim M.-H., Lee H.K. (2020). Matrix metalloproteinase 9-activatable peptide-conjugated hydrogel-based fluorogenic intraocular-lens sensor. Biosens. Bioelectron..

[20] Schwiegerling J. (2006). Recent developments in pseudophakic dysphotopsia. Curr. Opin. Ophthalmol..

[21] Masket S., Fram N.R. (2021). Pseudophakic dysphotopsia: Review of incidence, cause, and treatment of positive and negative dysphotopsia. Ophthalmology.

[22] Hu J., Sella R., Afshari N.A. (2018). Dysphotopsia: A multifaceted optic phenomenon. Curr. Opin. Ophthalmol..

[23] Bonsemeyer M.K.M., Becker E., Liekfeld A. (2021). Dysphotopsia and functional quality of vision after implantation of an intraocular lens with a 7.0 mm optic and plate haptic design. J. Cataract. Refract. Surg..

[24] Zopf D.A., Hollister S., Nelson M.E., Ohye R.G., Green G.E. (2013). Bioresorbable airway splint created with a three-dimensional printer. N. Engl. J. Med..

[25] Shiblee N.I., Ahmed K., Khosla A., Kawakami M., Furukawa H. (2018). 3D printing of shape memory hydrogels with tunable mechanical properties. Soft Matter.

[26] Hinze U., El-Tamer A., Reiß S., Stolz H., Guthoff R., Stachs O., Chichkov B.N. (2015). Implantatdesign mittels multiphotonen–polymerisation. Klin. Monatsbl. Augenheilkd..

[27] Chen X., Chen Z., Li Z., Zhao C., Zeng Y., Zou T., Fu C., Liu X., Xu H., Yin Z.Q. (2016). Grafted c-kit+/SSEA1− eye-wall progenitor cells delay retinal degeneration in mice by regulating neural plasticity and forming new graft-to-host synapses. Stem Cell Res. Ther..

[28] Norman J., Madurawe R.D., Moore C.M., Khan M.A., Khairuzzaman A. (2017). A new chapter in PAHrmaceutical manufacturing: 3D-printed drug products. Adv. Drug Deliv. Rev..

[29] Buwalda S.J., Vermonden T., Hennink W.E. (2017). Hydrogels for therapeutic delivery: Current developments and future directions. Biomacromolecules.

[30] Abdelkader H., Ismail S., Hussein A., Wu Z., Al-Kassas R., Alany R.G. (2012). Conjunctival and corneal tolerability assessment of ocular naltrexone niosomes and their ingredients on the hen’s egg chorioallantoic membrane and excised bovine cornea models. Int. J. Pharm..

[31] Li J.-W., Li Y.-J., Hu X.-S., Gong Y., Xu B.-B., Xu H.-W., Yin Z.-Q. (2020). Biosafety of a 3D-printed intraocular lens made of a poly(acrylamide-co-sodium acrylate) hydrogel in vitro and in vivo. Int. J. Ophthalmol..

[32] Shajari M., Kolb C.M., Petermann K., Böhm M., Herzog M., De’lorenzo N., Schönbrunn S., Kohnen T. (2018). Comparison of 9 modern intraocular lens power calculation formulas for a quadrifocal intraocular lens. J. Cataract. Refract. Surg..

[33] Eom Y., Song J.S., Kim H.M. (2016). Spectacle plane add power of multifocal intraocular lenses according to effective lens position. Can. J. Ophthalmol..

[34] Sennakesavan G., Mostakhdemin M., Dkhar L., Seyfoddin A., Fatihhi S. (2020). Acrylic acid/acrylamide based hydrogels and its properties—A review. Polym. Degrad. Stab..

[35] Liu Y.-C., Wong T.T., Mehta J.S. (2013). Intraocular lens as a drug delivery reservoir. Curr. Opin. Ophthalmol..

[36] Rim S.-B., Catrysse P.B., Dinyari R., Huang K., Peumans P. (2008). The optical advantages of curved focal plane arrays. Opt. Express.

[37] Rathnasamy G., Foulds W.S., Ling E.-A., Kaur C. (2019). Retinal microglia—A key player in healthy and diseased retina. Prog. Neurobiol..

[38] Pourjavadi A., Hosseinzadeh H. (2010). Synthesis and properties of partially hydrolyzed acrylonitrile-co-acrylamide superabsorbent hydrogel. Bull. Korean Chem. Soc..

[39] Siepser S.B., Wieland M. (1991). Animal model experimentation using the expansile hydrogel intraocular lens. J. Cataract. Refract. Surg..

[40] Ahmed M., Amirat M. (2024). FTIR, 1H, and 13C NMR Characterization and Antibacterial Activity of the Combination of Euphorbia Honey and Potato Starch. Comb. Chem. High Throughput Screen..

[41] Kumar R., Chaurasia A., Tewari A., Parashar A., Kumar R., Chaurasia A., Tewari A., Parashar A. (2024). Atomistic modelling and experimental study of tensile strength of nanocomposite hydrogel. Int. J. Mech. Sci..

[42] Ma H., Zou Y., Liu L., Zhang X., Yu J., Fan Y. (2024). Mussel-inspired chitin nanofiber adherable hydrogel sensor with interpenetrating network and great fatigue resistance for motion and acoustics monitoring. Int. J. Biol. Macromol..

[43] Ke W.-T., Cheng D.-Y., Wu I.-F., Liao Y.-C. (2024). 3D printed anti-swelling hydrogel scaffold with dialdehyde cellulose nanocrystals. Cellulose.

[44] (2009). Rubber, Vulcanized or Thermoplastic-Determination of Tensile Stress-Strain Properties.

[45] (2000). Plastics-Determination of Flexural Properties.

[46] (2011). Technical Specifications for Rubber Dies.

[47] (1992). Standard Test Method for ‘PLASTICS-Determination of Compressive Peoperties’.

[48] Madhi A., Shirkavand Hadavand B., Madhi A.H. (2024). Bio-friendly fluorescent polyvinyl alcohol/gelatin/chitosan hydrogel membranes strengthened by g-C3N4/CQDs nanocomposite: Preparation, investigation of UV-absorption, mechanical and rheological properties. Fuller. Nanotub. Carbon Nanostruct..

[49] Sheng L., Wang Y., Wang X., Han C., Chen Z. (2023). A thermal management strategy for electronic devices based on copper double skin inspired hydrogel. Int. J. Heat Mass Transf..

